# Comparative Cost Analysis of Surgical and PrePex Device Male Circumcision in Zimbabwe and Mozambique

**DOI:** 10.1097/QAI.0000000000000797

**Published:** 2016-05-24

**Authors:** Carl Schutte, M Tshimanga, Owen Mugurungi, Iotamo Come, Edgar Necochea, Mehebub Mahomed, Sinokuthemba Xaba, Debora Bossemeyer, Thais Ferreira, Lucinda Macaringue, Pessanai Chatikobo, Patricia Gundididza, Karin Hatzold

**Affiliations:** * Strategic Development Consultants, South Africa;; † ZICHIRE, Zimbabwe;; ‡ Ministry of Health and Child Care, Harare, Zimbabwe;; § Ministry of Health, Mozambique;; ‖ Jhpiego, Baltimore;; ¶ Jhpiego, Mozambique;; # PSI, Mozambique; and; ** Population Services International, Harare, Zimbabwe.

**Keywords:** voluntary medical male circumcision, PrePex device, Zimbabwe

## Abstract

**Background:**

The PrePex device has proven to be safe for voluntary medical male circumcision (VMMC) in adults in several African countries. Costing studies were conducted as part of a PrePex/Surgery comparison study in Zimbabwe and a pilot implementation study in Mozambique.

**Methods:**

The studies calculated per male circumcision unit costs using a cost–analysis approach. Both direct costs (consumable and nonconsumable supplies, device, personnel, associated staff training) and selected indirect costs (capital and support personnel costs) were calculated.

**Results:**

The cost comparison in Zimbabwe showed a unit cost per VMMC of $45.50 for PrePex and $53.08 for surgery. The unit cost difference was based on higher personnel and consumable supplies costs for the surgical procedure, which used disposable instrument kits. In Mozambique, the costing analysis estimated a higher unit cost for PrePex circumcision ($40.66) than for surgery ($20.85) because of higher consumable costs, particularly the PrePex device and lower consumable supplies costs for the surgical procedure using reusable instruments. Supplies and direct staff costs contributed 87.2% for PrePex and 65.8% for surgical unit costs in Mozambique.

**Discussion:**

PrePex device male circumcision could potentially be cheaper than surgery in Zimbabwe, especially in settings that lack the infrastructure and personnel required for surgical VMMC, and this might result in programmatic cost savings. In Mozambique, the surgical procedure seems to be less costly compared with PrePex mainly because of higher consumable supplies costs. With reduced device unit costs, PrePex VMMC could become more cost-efficient and considered as complementary for Mozambique's VMMC scale-up program.

## BACKGROUND

Following the recommendations from World Health Organization/Joint United Nations Programme on HIV/AIDS^1^ in 2007, 14 countries in East and Southern African have added voluntary medical male circumcision (VMMC) to their HIV prevention programs. Modeling suggests that scaling up VMMC in 13 Eastern and Southern African countries to 80% coverage over 5 years, assuming a total of 20.3 million male circumcisions (MCs), and maintaining that coverage through 2025 could avert 3.4 million HIV infections over that period and save $16.5 billion in treatment costs.^2^ Progress to reach the 80% coverage target has varied by country and has not been as fast as expected. According to recent estimates, it is expected that a total of 10 million men will have been circumcised by the end of 2015 since the first national VMMC programs started in 2007.^3^

It has been hypothesized that MC devices, in particular the PrePex device, could increase efficiency and effectiveness of MC scale-up through reduced time required for procedures, leading to greater throughput of clients and reduced cost per procedure. The PrePex device is an elastic collar compression device that works through slow compression of the foreskin producing tissue devitalization and necrosis.^4^ PrePex MC seems easier to perform and allows for task shifting to nonphysician providers and even primary health care nurses. Because of its advantages from the client perspective (a nonsurgical procedure that does not require injectable anesthesia and that allows for early resumption of work/return to school), device MC might also lead to improved acceptability among males. Nevertheless, there are also disadvantages of using the device including longer healing times, pain while wearing the device and at removal, and odor.^5^

A series of 3 studies of the PrePex device completed in Rwanda^6–8^ were followed by 3 independent PrePex device studies in Zimbabwe.^9–11^ Following the World Health Organization recommendations in the Framework for Clinical Evaluation of Devices for MC, several countries have undertaken pilot implementation studies to demonstrate the feasibility and acceptability of the new device.^4,12^ The PrePex device costing studies conducted in Kenya,^13^ Uganda,^14^ Zimbabwe,^15^ and other countries have not shown that PrePex device MC results in increased cost-efficiency.

## METHODS

Cost data were collected as part of a comparison trial (PrePex versus forceps-guided surgery) in Zimbabwe (November 2011–August 2012) and a PrePex field study in Mozambique (July–November 2013). The comparison trial in Zimbabwe involved 240 individuals aged 18–49; 80 were allocated randomly to surgery and 160 to the device. The field study in Mozambique had a sample size of 504 adult men (18–49 years), all of whom were circumcised with the PrePex device. A smaller sample of 32 surgical circumcisions using the forceps-guided method was used to collect costing data to estimate the unit costs for surgical circumcision in Mozambique. In both studies, the primary objective was to calculate the unit costs associated with PrePex MC and to compare these with the unit cost of standard surgical circumcision.

This costing analysis excluded client costs (transport, absenteeism from work, and caregiver costs). All costs were presented in 2011/2012 constant US dollars. Since Zimbabwe officially adopted the US dollar as its principal currency in 2009, the analysis in Zimbabwe assumed an exchange rate of US$1 = US$1. For the cost analysis in Mozambique, prevailing exchange rates were used. The Decision Makers Program Planning Tool was adapted to calculate the unit cost of VMMC.^16^

Information on the costs of supplies was accessed from invoices and receipts sourced from the research partner, procurement, and accounts departments. A structured survey tool was used to collect information on the consumption of commodities and preprocedure and postprocedure counseling times. Time to perform procedures and follow-up visits was recorded. The time for preparation of the client, penile measurement, all steps of the device placement and removal, and wound dressing after device removal was recorded for the PrePex procedure. For the surgical procedure, time for skin disinfection, injection of local anesthesia, and all steps of the procedure including wound dressing were recorded. Three routine follow-up visits were assumed for both surgical and PrePex (after device removal) circumcision in the Zimbabwe study, whereas 2 routine follow-up visits were assumed after surgical circumcision and no routine follow-up visits were included after removal of the PrePex device in Mozambique.

### Assumptions Site Capacity Utilization

#### Zimbabwe Costing Model

Assuming that 2 circumcision teams of 3 nurses in each team supervised by one doctor would work for 5 days per week and 48 weeks in the year, performing together 40 circumcisions a day, the site capacity for surgical MCs in Zimbabwe was estimated at 9600 circumcisions per year. The estimated annual output for device-based circumcisions was based on the “time spent per circumcision” recorded during the study. Time spent per circumcision included all steps of the procedure from skin preparation to the client leaving the operating table. Assuming an average of 30 PrePex placements per day per team, the annual capacity for device circumcisions was estimated at 14,400 MCs per year for 2 provider teams composed each of 2 nurse operators, 1 nurse counselor, and 1 supervising doctor for 2 teams of nurses.

#### Mozambique Costing Model

In the Mozambique model, it was assumed that 7350 surgical circumcisions could be achieved per year at a static site in Maputo, assuming that 2 circumcision teams would be working for 49 weeks in the year, providing services on 5 days per week and performing 30 circumcisions a day. The surgical circumcision model provided for the cost of 1 registered nurse (RN) and 1 ancillary worker assisting the nurse. For the PrePex model, both procedure staff were RNs. Introducing PrePex circumcisions was assumed to result in a 10% increase in VMMC uptake, resulting in a total of 8085 device MCs per year.

#### Direct Costs

##### Supplies and Device Costs

For both studies, an ingredients-based bottom-up approach was used to calculate circumcision costs at the facility level. Based on data collected, the rate of consumption of each item was determined. Based on program data from both countries, consumables included rapid HIV test kits for 90% of clients. The cost per device was included at $18 in Zimbabwe and at $24 in Mozambique. In Zimbabwe, disposable MC kits costs were included at $19.52 each. A provision for supply chain management equal to 20% of consumables unit costs was assumed in the costing. This assumption was based on previous VMMC costing studies.^17^

#### Personnel Costs

The total unit cost contribution of personnel to each procedure was derived by summing up costs of time spent to conduct the MC procedure for each provider. The time contribution of the different providers to an MC procedure was multiplied by hourly salaries to derive the personnel unit cost. In both countries, the salaries paid during the study were used for costing clinical staff time. These were significantly higher than government pay rates in Zimbabwe but approximated Ministry of Health (MOH) pay rates in Mozambique. Costs included supervision of the circumcision teams by a surgical technician, staff time for HIV testing and counseling (HTC), and support staff. The costing models included nurses as providers for both surgery and PrePex device procedures. Training costs were based on actual provider training expenditure amortized over a 5-year period in line with turnover rates of health care workers in Zimbabwe and Mozambique.

#### Capital Costs

Capital costs of equipment were annualized using the assumed useful life years and were divided by the annual number of circumcisions assumed for each facility. With the exception of the equipment in the device procedure rooms, all equipment was shared between surgical and device services. Capital items excluded built infrastructure but included a rental cost for the MC facilities (Zimbabwe only) and ongoing renovations and repairs. In Mozambique, an average up-front cost for site renovations was included in the list of capital items.

## RESULTS

Table 1 shows the drivers for the PrePex and surgical circumcision unit cost in the Zimbabwe and Mozambique studies. In Zimbabwe, the estimated total unit cost was $45.50 and $53.08 per circumcision for the PrePex device and surgical circumcisions, respectively, whereas in Mozambique, the estimated total unit cost was $40.66 and $20.85 per circumcision for the PrePex device and surgical circumcisions, respectively. Consumables and direct staff costs contributed more than 90% of the unit cost for both surgery and the PrePex device in Zimbabwe and 69.3% for surgery and 86% for PrePex in Mozambique.

**TABLE 1.**
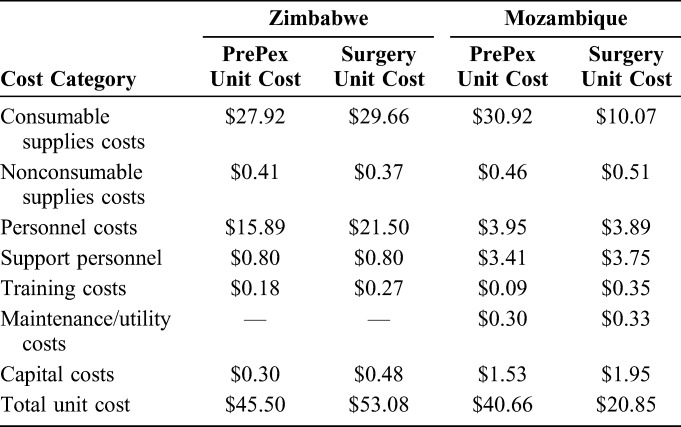
Cost Components of Unit Cost PrePex and Surgery, Zimbabwe and Mozambique

### Consumables Costs

The largest unit cost driver was consumables estimated at $27.92 (Zimbabwe) and $30.92 (Mozambique) for device MC and $29.66 (Zimbabwe) and $10.07 (Mozambique) for surgical circumcision. For PrePex MC, the largest commodity cost was the device itself at $18.00 and $24.00 in Zimbabwe and Mozambique, respectively. In Zimbabwe, the surgical disposable kit was the largest contributor to the commodities costs for surgery at $19.56. Other items comprised pharmaceutical (local injectable anesthesia for surgery and local anesthetic cream for PrePex) and nonpharmaceutical consumables, sutures for surgical procedures in Mozambique, sterilizing scrub and solution, gloves, linens, gauze pads, bandages, and HIV rapid test kits.

### Personnel Costs

The second largest contributor to the unit costs in both country studies was personnel costs. In Zimbabwe, personal costs were $16.38 and $22.69 per MC for the device and surgery, respectively. In the Mozambique study, personnel costs were estimated at $3.95 for the PrePex and $3.89 for surgical MC.

PrePex clients had to attend a PrePex-specific counseling session in addition to VMMC counseling, which doubled the amount of counseling time invested by the provider. Furthermore, for PrePex, additional personnel time was included for client screening. In Zimbabwe, time for HTC contributed 48.8% and 35.3% to the total personnel costs for the PrePex and surgery, respectively. In Mozambique, HTC contributed to 19.8% and 19.5% of the personnel component unit cost for surgery and PrePex, respectively.

In the Mozambique study, the estimated costs of support staff at $3.41 for device and $3.75 for surgical circumcision, respectively, were almost as high as those for provider costs. Support staff included a surgical technician for supervision, a receptionist, and a dedicated ancillary worker.

### Other Cost Components

In both countries, training costs for both surgery and PrePex were based on latest actual expenditure incurred by the programs. Because of the higher annual capacity/site utilization assumed for PrePex device circumcisions, the training cost per MC unit was estimated to be lower for the device method ($0.18 versus $0.27 for surgery) in Zimbabwe. In Mozambique, the per MC training cost was estimated at $0.09 for PrePex and $0.35 for surgery.

## DISCUSSION

The unit cost of an MC using the PrePex device was estimated at $44.99 against $54.26 for forceps-guided surgery in the Zimbabwe study, whereas unit costs in Mozambique were estimated at $40.66 and $20.85 for PrePex and surgery, respectively. The per MC unit costs for PrePex in the costing studies presented here compare well with results from other recent PrePex costing studies conducted in Kenya^13^ ($44.5); and are slightly higher than those estimated in a study from Uganda^14^ ($30.55).

The most sensitive cost categories were consumable supplies and direct personnel costs. In the study in Zimbabwe, consumable supplies costs and personnel costs together contributed to 96% of the PrePex and surgical MC unit costs. In Mozambique, these 2 cost categories contributed to 86% and 63% of the unit costs for PrePex and surgery. The largest cost contributor to the PrePex unit cost in both studies was the consumable supply cost and was mainly influenced by the device cost itself. Any changes in the device cost would have a direct impact on the unit cost. Negotiations to lower unit costs present an opportunity for substantial cost reduction for the PrePex procedure.

The main difference contributing to the higher MC unit costs for PrePex as compared with surgery in the Mozambique study was also based on consumable costs. The relatively low consumables unit cost for surgery was influenced by the use of reusable surgical instruments with much lower consumable supplies costs ($10.07) as compared with PrePex ($30.92).

Although both costing studies found lower personnel cost for the actual PrePex procedure as compared with surgery based on reduced procedure time, this higher efficiency was offset in the Mozambique study by the fact that 2 RNs conducted the PrePex procedure, whereas for surgery, an ancillary provider assisted the nurse provider. In addition, as part of the standard procedure, PrePex clients had to attend standard VMMC counseling and PrePex-specific counseling, nearly doubling the amount of counseling time. During the PrePex study, individual clinical screening time was recorded as nearly 10 times as long as for surgery. It is assumed that these inefficiencies would be addressed with increasing experience with the device in routine service delivery. Frequency of follow-up visits for both surgery and PrePex differed substantially between both 2studies and this impacted on personnel cost contribution to unit costs.

Because counseling contributes largely to the personnel costs, the shorter procedure time for PrePex does not seem to have a major impact on the personnel costs. The differences in provider pay rates in Zimbabwe and Mozambique, given the substantial contribution of personnel costs to overall costs, makes comparison between both 2countries difficult. Costing data from these studies are thus not generalizable and are only appropriate in the context of the countries in which the studies were conducted.

Previous studies found that 5%–18% of adult MC clients were ineligible for PrePex because of phimosis or tight foreskin and would require surgery using the standard forceps-guided or dorsal slit method.^17^ Currently, approximately 70% of VMMC clients in Zimbabwe and Mozambique are below the age of 18 years and PrePex is only prequalified for use in adults aged above 18 years.^5^ Alternative options and referral systems would need to be provided to ensure that most males presenting for VMMC could be served. These mechanisms would likely increase the cost for delivering VMMC services. Any VMMC scale-up costing process should take into account the proportion of males in the target group who require surgical circumcision as this has significant impact on operational logistics, implementation planning, and costs.^15^

### Limitations of the Study

The costing studies in Zimbabwe and Mozambique were conducted at fixed VMMC locations at high-volume urban sites and extrapolation of the findings to low-volume rural sites or mobile VMMC models cannot be done. The methodologies and assumptions in both country studies were very different, which makes any comparison between the unit costs in each of the countries difficult. Limited sensitivity analysis made it difficult to understand the impact varying inputs have on the unit costs.

Direct staff time was calculated on the basis of time spent on each circumcision, excluding time lost between clients. This assumption and approach is valid in an integrated VMMC setting in which providers have duties other than VMMC. The approach also assumes a certain level of client throughput and site capacity utilization. If providers would be deployed to only deliver VMMC services and where the assumed throughput of circumcisions is not achieved, the unit costs per circumcision would increase substantially.

The costs were based on the current local prices that are subject to inflation and exchange rate variations. The studies did not evaluate the unit cost of demand creation, which might be an important cost factor in the unit cost analysis. Cost of surgical kits and device disposal included sharps boxes and biohazard trash bags but excluded capital costs associated with the procurement of additional incinerators or employment of additional waste management staff. With further scale-up, waste management will add to the unit cost of circumcision, and existing research indicates that these costs may be significant.^17^ The unit costs presented in this report cannot be compared without adjustments to unit costs derived from actual program implementation.

## CONCLUSION

The PrePex device could potentially reduce the per MC unit cost in the VMMC program in Zimbabwe because of increased efficiency of the procedure resulting in lower personnel costs and potentially higher site capacity utilization. In contrast, the cost comparison from Mozambique showed that estimated MC unit costs for surgery were substantially lower because of much lower consumable supplies costs for surgery. Nevertheless, if incorporating PrePex would indeed contribute to maximizing VMMC throughput, adding PrePex to surgical VMMC could potentially increase cost-efficiency of current programs. Collection and analysis of actual PrePex VMMC program cost data will help countries to make program decisions regarding the incorporation of PrePex into their VMMC scale-up plans.
